# Comparison of retinal thickness measurements among four different optical coherence tomography devices

**DOI:** 10.1038/s41598-024-54109-6

**Published:** 2024-02-12

**Authors:** Ki Tae Nam, Cheolmin Yun, Myungho Seo, Somin Ahn, Jaeryung Oh

**Affiliations:** 1 Department of Ophthalmology, Jeju National University College of Medicine, Jeju, South Korea; 2 Department of Ophthalmology, Korea University College of Medicine, Seoul, South Korea

**Keywords:** Eye diseases, Retina

## Abstract

We sought to compare the retinal thickness measurements collected using different optical coherence tomography (OCT) devices. This prospective study included 21 healthy cases, and the retinal thickness was measured using the PLEX Elite (Carl Zeiss Meditec, Dublin, California, USA), DRI OCT-1 Atlantis (Topcon Corp, Tokyo, Japan), Cirrus 5000 HD-OCT (Carl Zeiss Meditec), and Spectralis OCT (Heidelberg Engineering, Heidelberg, Germany), respectively. The mean central retinal thickness (CRT) and mean retinal thickness of the Early Treatment of Diabetic Retinopathy Study (ETDRS) area were compared. The CRT varied significantly among the different OCT devices (*P* < 0.001). Post-hoc analysis revealed that the CRT measured using PLEX Elite (278.95 ± 20.04 µm) and Spectralis (271.86 ± 17.92 µm) were similar, and both were greater than the CRT measurements of DRI OCT-1 (239.57 ± 21.06 µm) and Cirrus (256.76 ± 17.82 µm). Additionally, the mean retinal thickness in each ETDRS area showed significant differences among the four devices (all *P* < 0.001). The mean retinal thickness measured varied according to the device used, and this needs to be considered when comparing retinal thickness measurements taken with different devices.

## Introduction

Advances in optical coherence tomography (OCT) technology have led to the development of devices that use different algorithms. Spectral-domain OCT (SD-OCT) provides faster imaging, higher-resolution images, and an improved signal-to-noise ratio that allows us to distinguish between different layers of the retina^1^. Swept-source OCT (SS-OCT) increases the acquisition rate of conventional SD-OCT from 20,000 to 40,000 A-scans per second to 100,000–400,000 A-scans per second and uses a longer wavelength (up to 1050 nm) to provide very high spatial resolution^2^. Various OCT devices have shown high reproducibility in distinguishing each layer of the retina in detail and accurately presenting the thickness of the entire retina, including the thickness of each layer^3,4^. Retinal thickness is also useful for evaluating the course of disease in conditions that cause macular edema, such as diabetic macular edema, retinal vein occlusion, central serous chorioretinopathy, and exudative age-related macular degeneration.

OCT images that observe the structure of the retina may differ in their retinal thickness measurements due to variations in the wavelengths used by the devices and image processing algorithms^5–8^. In addition, it has been reported that significant differences in retinal thickness between OCT devices and analytical methods in diseases that cause changes in retinal thickness also exist, and these measurements are not interchangeable, which should be noted during application in clinical practice^5,9–11^. Most existing studies compared two different types of devices or included patients with macular edema, which can affect retinal thickness acquisition, so there are limitations in comparing differences in overall retinal thickness among different devices.

Recently, retinal thickness measurements made using OCT angiography (OCTA) from the SS-OCT device (PLEX Elite; Carl Zeiss Meditec, Dublin, California, USA) have become available for clinical use. However, it has not yet been reported whether PLEX Elite can be used to measure retinal thickness using OCTA images and whether its results are interchangeable with those of other OCT devices traditionally used to measure retinal thickness. Therefore, we aimed to compare retinal thickness values in healthy adults collected using four different devices (PLEX Elite, DRI OCT-1 Atlantis [Topcon Corp, Tokyo, Japan], Cirrus 5000 HD-OCT [Carl Zeiss Meditec] and Spectralis [Heidelberg Engineering, Heidelberg, Germany]).

## Results

A total of 21 healthy volunteers (21 eyes) were included, with a mean age of 33.57 ± 4.02 (range, 30–45) years. Eleven (52%) patients were women, and the mean axial length was 25.33 ± 0.66 mm.

**Figure 1:**
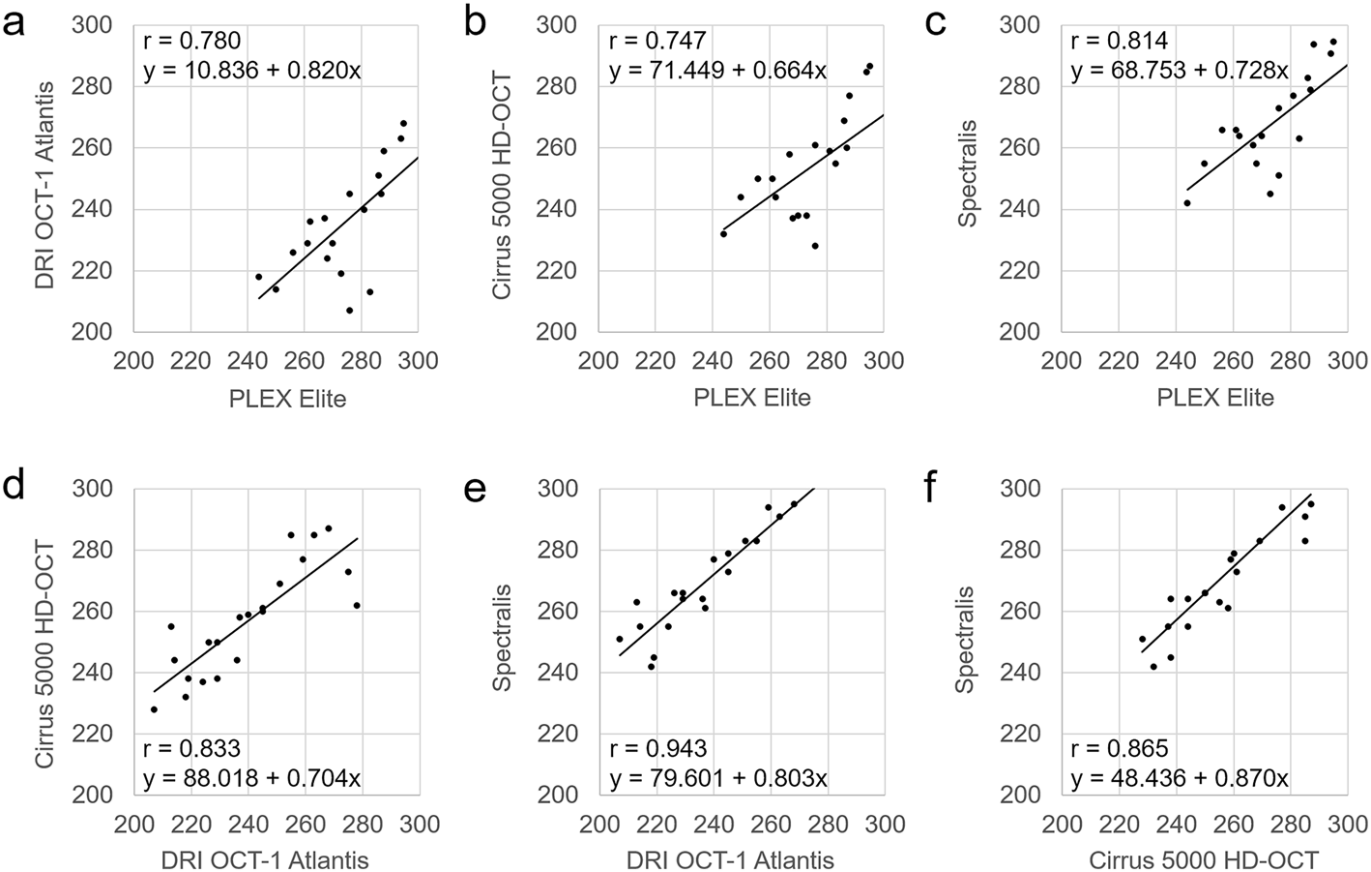
Pearson correlation coefficients and regression equations from different devices for measuring central retinal thickness (µm). (**a**) PLEX Elite and DRI OCT-1 Atlantis. (**b**) PLEX Elite and Cirrus 5000 HD-OCT. (**c**) PLEX Elite and Spectralis. (**d**) DRI OCT-1 Atlantis and Cirrus 5000 HD-OCT. (**e**) DRI OCT-1 Atlantis and Spectralis. (**f**) Cirrus 5000 HD-OCT and Spectralis.

**Figure 2:**
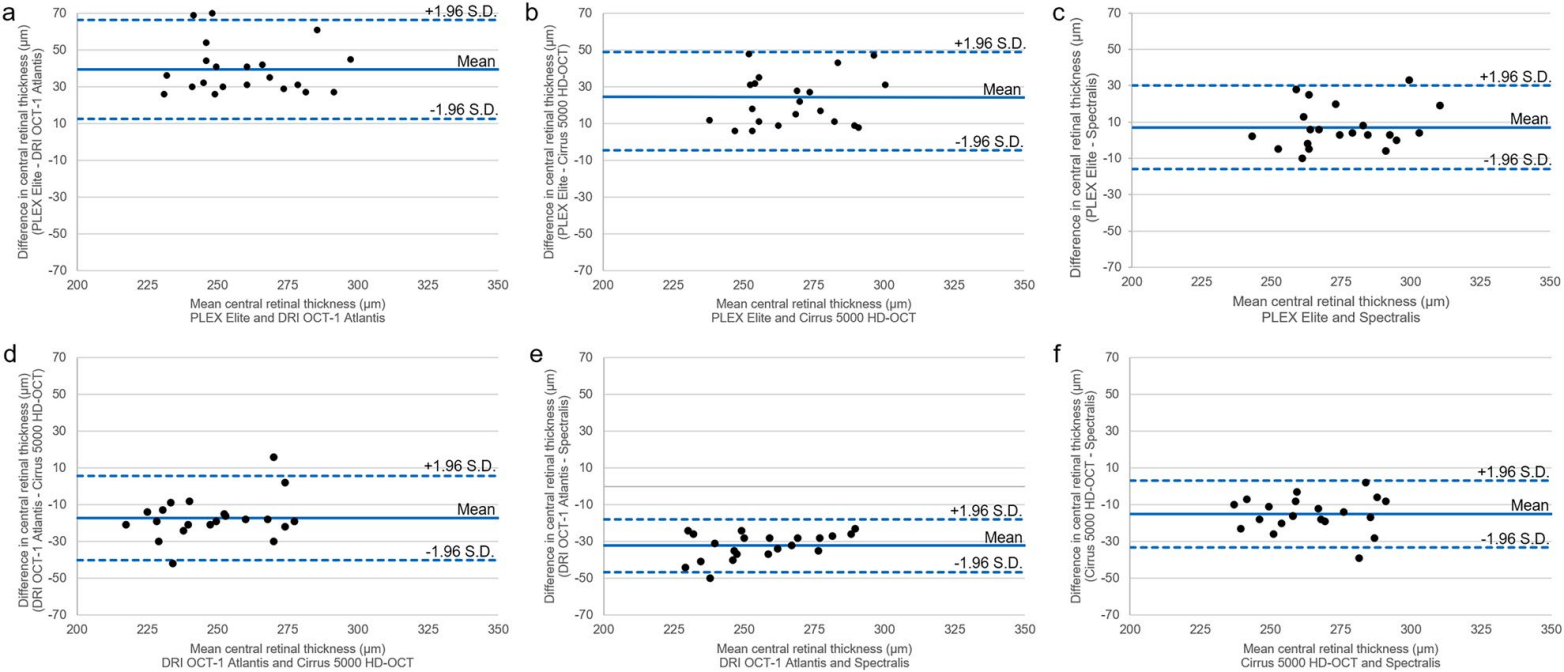
Bland–Altman plots comparing central retinal thickness measurements from different devices. The solid line represents the mean of the two measurements, while the dashed line indicates the 95% limits of agreements. (**a**) Comparison of PLEX Elite and DRI OCT-1 Atlantis. (**b**) Comparison of PLEX Elite and Cirrus 5000 HD-OCT. (**c**) Comparison of PLEX Elite and Spectralis. (**d**) Comparison of DRI OCT-1 Atlantis and Cirrus 5000 HD-OCT. (**e**) Comparison of DRI OCT-1 Atlantis and Spectralis. (**f**) Comparison of Cirrus 5000 HD-OCT and Spectralis.

The central retinal thickness (CRT) exhibited a statistically significant difference among the four devices (PLEX Elite, 278.95 ± 20.04 µm; DRI OCT-1 Atlantis, 239.57 ± 21.06 µm; Cirrus 5000 HD-OCT, 256.76 ± 17.82 µm; Spectralis, 271.86 ± 17.92 µm; *P* < 0.001) (Table 1). Based on the post hoc analysis, there was only no statistically significant difference between PLEX Elite and Spectralis. The CRT showed a statistically significant positive correlation any pair of the four devices (all *P* < 0.001) (Fig. 1, Table 2). the intraclass correlation coefficient (ICC)s of CRT between PLEX Elite and the other devices, respectively, ranged from 0.852 to 0.894 (Table 3). Bland–Altman plots comparing CRT from the four devices revealed the following mean differences and 95% limits of agreement: 39.38 ± 26.78 µm, PLEX Elite vs. DRI OCT-1 Atlantis; 22.19 ± 26.70 µm, PLEX Elite vs. Cirrus 5000 HD-OCT; 7.10 ± 23.02 µm, PLEX Elite vs. Spectralis; − 17.19 ± 22.87 µm, DRI OCT-1 Atlantis vs. Cirrus 5000 HD-OCT; − 32.29 ± 14.23 µm, DRI OCT-1 Atlantis vs. Spectralis; and − 15.10 ± 18.19 µm, Cirrus 5000 HD-OCT vs. Spectralis (Fig. 2).

**Table 1:** Comparison of retinal thickness (μm) measurements among four different devices.

	PLEX Elite	DRI OCT-1 Atlantis	Cirrus 5000 HD-OCT	Spectralis	*P* value*
Center	278.95 ± 20.04^a^	239.57 ± 21.06^b^	256.76 ± 17.82^c^	271.86 ± 17.92^a^	< 0.001
Inner					
Superior	344.43 ± 12.25^a^	313.52 ± 11.22^b^	327.95 ± 11.00^c^	347.95 ± 9.40^a^	< 0.001
Temporal	325.38 ± 12.20^a^	302.05 ± 9.77^b^	315.00 ± 10.23^c^	332.91 ± 10.38^d^	< 0.001
Inferior	334.29 ± 13.58^a^	309.43 ± 10.83^b^	320.76 ± 9.49^c^	341.57 ± 10.55^d^	< 0.001
Nasal	344.86 ± 11.29^a^	313.95 ± 12.12^b^	328.67 ± 9.15^c^	348.14 ± 11.21^a^	< 0.001
Outer					
Superior	295.33 ± 12.26^a^	274.62 ± 9.66^b^	284.10 ± 9.43^c^	301.62 ± 9.52^d^	< 0.001
Temporal	278.62 ± 17.19^a^	256.57 ± 10.90^b^	265.24 ± 10.82^c^	290.62 ± 22.73^a^	< 0.001
Inferior	279.81 ± 12.59^a^	255.71 ± 11.25^b^	264.71 ± 11.17^c^	283.52 ± 10.82^a^	< 0.001
Nasal	311.57 ± 13.30^a^	289.29 ± 11.71^b^	302.24 ± 9.80^c^	318.05 ± 11.12^d^	< 0.001

Values are shown as mean ± standard deviation (µm).

*Repeated-measures analysis of variance; a, b, c, d: mean values followed by the same letter do not differ significantly from one another according to post hoc analysis.

**Table 2:** Pearson correlation coefficients of retinal thickness measurements from four different optical coherence tomography devices.

	PLEX Elite and DRI OCT-1 Atlantis	PLEX Elite and Cirrus 5000 HD-OCT	PLEX Elite and Spectralis	DRI OCT-1 Atlantis and Cirrus 5000 HD-OCT	DRI OCT-1 Atlantis and Spectralis	Cirrus 5000 HD-OCT and Spectralis
Center	0.780 (*P* < 0.001)	0.747 (*P* < 0.001)	0.814 (*P* < 0.001)	0.833 (*P* < 0.001)	0.943 (*P* < 0.001)	0.865 (*P* < 0.001)
Inner						
Superior	0.712 (*P* < 0.001)	0.592 (*P* = 0.005)	0.728 (*P* < 0.001)	0.315 (*P* = 0.164)	0.938 (*P* < 0.001)	0.381 (*P* = 0.088)
Temporal	0.721 (*P* < 0.001)	0.676 (*P* = 0.001)	0.629 (*P* = 0.002)	0.701 (*P* < 0.001)	0.882 (*P* < 0.001)	0.592 (*P* = 0.005)
Inferior	0.669 (*P* = 0.001)	0.566 (*P* = 0.007)	0.725 (*P* < 0.001)	0.767 (*P* < 0.001)	0.916 (*P* < 0.001)	0.782 (*P* < 0.001)
Nasal	0.855 (*P* < 0.001)	0.791 (*P* < 0.001)	0.878 (*P* < 0.001)	0.697 (*P* < 0.001)	0.839 (*P* < 0.001)	0.611 (*P* = 0.003)
Outer						
Superior	0.794 (*P* < 0.001)	0.599 (*P* = 0.004)	0.775 (*P* < 0.001)	0.775 (*P* < 0.001)	0.931 (*P* < 0.001)	0.781 (*P* < 0.001)
Temporal	0.583 (*P* = 0.006)	0.657 (*P* = 0.001)	0.342 (*P* = 0.129)	0.869 (*P* < 0.001)	0.461 (*P* = 0.036)	0.293 (*P* = 0.198)
Inferior	0.771 (*P* < 0.001)	0.825 (*P* < 0.001)	0.771 (*P* < 0.001)	0.865 (*P* < 0.001)	0.955 (*P* < 0.001)	0.827 (*P* < 0.001)
Nasal	0.849 (*P* < 0.001)	0.835 (*P* < 0.001)	0.869 (*P* < 0.001)	0.803 (*P* < 0.001)	0.932 (*P* < 0.001)	0.823 (*P* < 0.001)

**Table 3:** Intraclass correlation coefficient (95% confidence interval) values of retinal thickness measurements from four different optical coherence tomography devices.

	PLEX Elite and DRI OCT-1 Atlantis	PLEX Elite and Cirrus 5000 HD-OCT	PLEX Elite and Spectralis	DRI OCT-1 Atlantis and Cirrus 5000 HD-OCT	DRI OCT-1 Atlantis and Spectralis	Cirrus 5000 HD-OCT and Spectralis
Center	0.876 (0.694–0.950)	0.852 (0.635–0.940)	0.894 (0.740–0.957)	0.902 (0.758–0.960)	0.964 (0.912–0.986)	0.928 (0.822–0.971)
Inner						
Superior	0.830 (0.581–0.931)	0.741 (0.362–0.895)	0.826 (0.571–0.929)	0.479 (–0.283–0.789)	0.960 (0.902–0.9984)	0.547 (–0.116–0.816)
Temporal	0.826 (0.571–0.929)	0.799 (0.505–0.919)	0.766 (0.423–0.905)	0.824 (0.565–0.928)	0.936 (0.843–0.974)	0.744 (0.369–0.896)
Inferior	0.790 (0.482–0.915)	0.694 (0.247–0.876)	0.825 (0.569–0.929)	0.864 (0.665–0.945)	0.956 (0.891–0.982)	0.875 (0.692–0.949)
Nasal	0.921 (0.804–0.968)	0.872 (0.686–0.948)	0.935 (0.839–0.974)	0.803 (0.514–0.920)	0.911 (0.780–0.964)	0.749 (0.382–0.898)
Outer						
Superior	0.871 (0.682–0.948)	0.734 (0.343–0.892)	0.858 (0.649–0.942)	0.873 (0.688–0.949)	0.964 (0.912–0.985)	0.877 (0.697–0.950)
Temporal	0.690 (0.237–0.874)	0.744 (0.370–0.896)	0.496 (–0.243–0.795)	0.930 (0.828–0.972)	0.528 (–0.162–0.809)	0.370 (–0.552–0.744)
Inferior	0.867 (0.673–0.946)	0.900 (0.755–0.960)	0.865 (0.667–0.945)	0.928 (0.822–0.971)	0.977 (0.942–0.990)	0.905 (0.765–0.961)
Nasal	0.914 (0.789–0.965)	0.887 (0.723–0.954)	0.922 (0.808–0.968)	0.883 (0.712–0.953)	0.964 (0.912–0.986)	0.899 (0.752–0.959)

The mean retinal thickness of the Early Treatment Diabetic Retinopathy Study (ETDRS) area, aside from the CRT, also exhibited statistically significant differences among the four devices (all *P* < 0.001) (Table 1). Based on the post hoc analysis, the mean retinal thickness in the inner superior, inner nasal, outer temporal, and outer inferior areas was not statistically significantly different between PLEX Elite and Spectralis. The mean retinal thickness in each ETDRS area showed positive correlations any pair of the four devices with the following exceptions (inner superior macular thickness zone, Cirrus 5000 HD-OCT and DRI OCT-1 Atlantis, Cirrus 5000 HD-OCT and Spectralis, respectively; outer temporal macular thickness zone: Spectralis and PLEX Elite, Spectralis and Cirrus 5000 HD-OCT, respectively) (Table 2). The ICCs of retinal thickness between PLEX Elite and the other devices ranged from 0.496 to 0.935 (Table 3).

## Discussion

This study analyzed the retinal thickness in healthy adults using four different devices (PLEX Elite, DRI OCT-1 Atlantis, Cirrus 5000 HD-OCT, and Spectralis) and found that there were differences in retinal thickness measured in the same individuals. The CRT measurement was thickest when using PLEX Elite and Spectralis, with no statistically significant difference between these two devices, followed by Cirrus 5000 HD-OCT and DRI OCT-1 Atlantis. The other ETDRS area showed similar trends in retinal thickness according to the device used.

CRT has been used as a marker to assess activity or treatment efficacy in various retinal diseases. However, a drawback is that CRT values can differ depending on the machine used for measurement, making them non-interchangeable between different devices. This has made it challenging to consider CRT measured on one machine as equivalent to CRT measured on another machine in real clinical settings. Consequently, numerous studies have been conducted in the past to address these differences, and clinicians have had to take into account the variations in CRT values measured by different devices when making clinical decisions (Supplementary Table S1)^5,9,12–17^.

**Figure 3:**
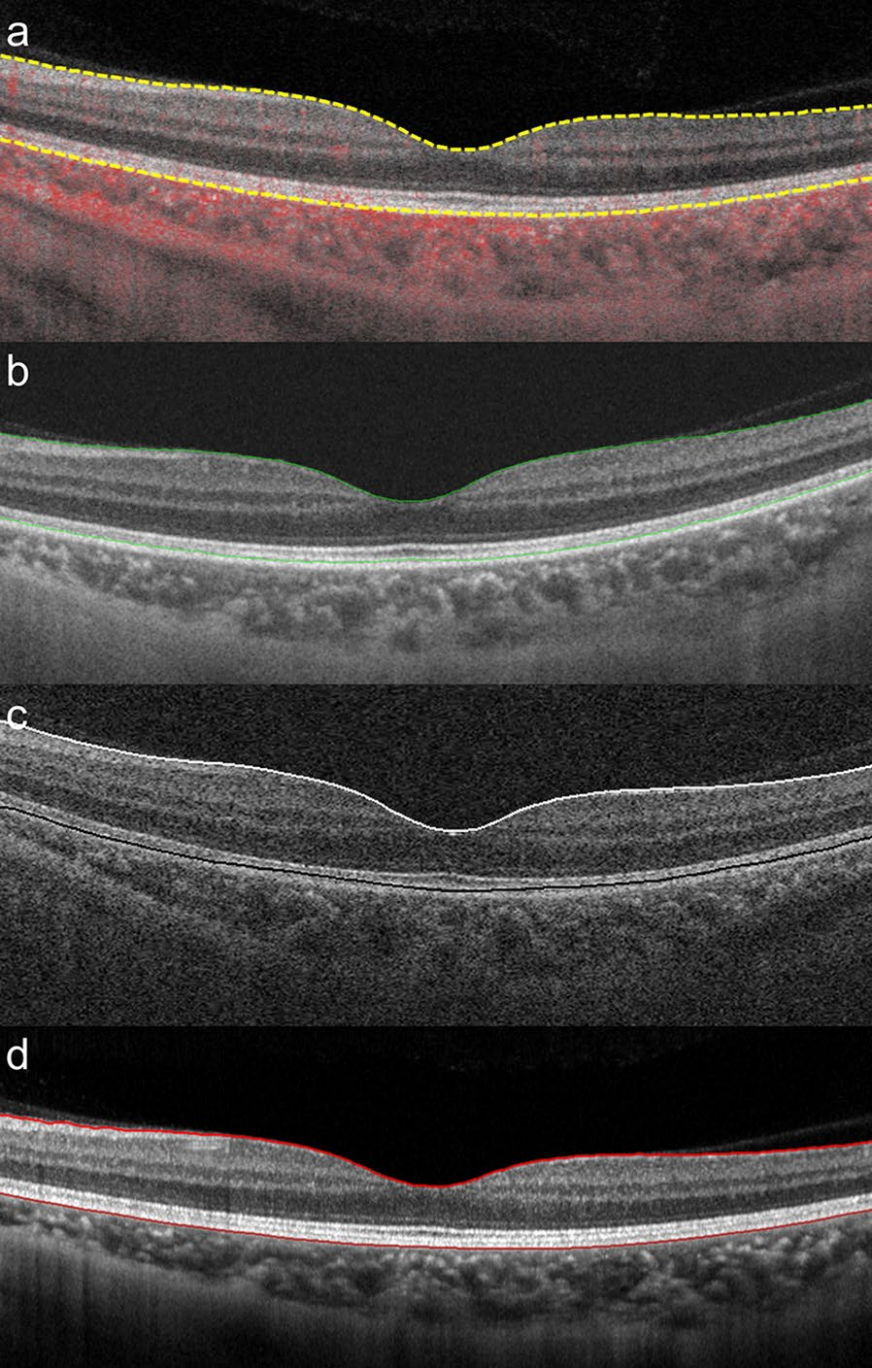
Optical coherence tomography (OCT) segmentation boundaries of retinal thickness according to different OCT devices. (**a**) Segmentation boundaries of PLEX Elite, with the lower boundary at Bruch’s membrane (BM) (dotted yellow line). (**b**) Segmentation boundaries of DRI OCT-1 Atlantis, with the lower boundary at the border of the outer segment of photoreceptor and retinal pigment epithelium (RPE) (solid green line). (**c**) Segmentation boundaries of Cirrus 5000 HD-OCT, with the lower boundary at the middle of the RPE (solid black line). (**d**) Segmentation boundaries of Spectralis, with the lower boundary at BM (solid red line).

Based on the results of this study, the mean CRT of healthy adults assessed using PLEX Elite was 278 µm and that of healthy adults assessed using DRI OCT-1 Atlantis was 239 µm, for a difference of approximately 40 µm. Such differences are thought to be because the anatomical basis for measuring thickness varies between devices. PLEX Elite and Spectralis measure the distance from the internal limiting membrane (ILM) to Bruch’s membrane (BM), while Cirrus 5000 HD-OCT measures from the ILM to the middle layer of the retinal pigment epithelium (RPE) and DRI OCT-1 Atlantis measures from the ILM to the border of the outer photoreceptor segment (OS) and RPE (Table 4, Fig. 3)^5^. Due to these differences, there was variation in CRT measurements among devices. However, CRT values obtained using DRI OCT-1 and Spectralis showed a higher correlation coefficient and narrower 95% agreement limit on the Bland–Altman plot compared with other device comparisons. This suggests that, despite differences in measurements, CRT values obtained from these two devices are likely more interchangeable than values from other devices.

**Table 4:** Characteristics of optical coherence tomography devices.

Device	Methodology	Wavelength (nm)	Axial/transverse resolution (μm)	Pixels	Anatomic basis of retinal thickness
PLEX Elite	Swept source	1060	6.3/20	1024	ILM BM
DRI OCT-1 Atlantis	Swept source	1050	5/15	320	ILM OS/RPE boundary
Cirrus 5000 HD-OCT	Spectral domain	840	5/15	412	ILM Middle of RPE
Spectralis	Spectral domain	870	7/14	512	ILM BM

BM, Bruch's membrane; ILM, internal limiting membrane; OS, outer segment of photoreceptor; RPE, retinal pigment epithelium.

Tan et al. reported retinal thicknesses of 238 µm, 271 µm, and 254 µm in adults measured using DRI OCT-1 Atlantis, Spectralis, and Cirrus HD-OCT, respectively^5^. These authors reported that this finding is due to differences in the lower segment boundary of an anatomic basis. This is consistent with our results and given that the CRT of PLEX Elite is not statistically different from that of Spectralis in our study, it can be interpreted that the value of CRT is due to the anatomical basis itself rather than other characteristics of the device (swept-source versus spectral-domain OCT; differences in wavelength, resolution, and pixel count).

Tepelus et al. compared CRT measurements using two different SD-OCT systems, RS-3000 Advance (Nidek) and Cirrus HD-OCT (Carl Zeiss), in patients with dry age-related macular degeneration, with RS-3000 Advance measuring 257 µm and Cirrus HD-OCT measuring 238 µm^4^. The anatomical range for measuring CRT using these two instruments was from the ILM to the outer border of the RPE for the RS-3000 Advance and from the ILM to the middle layer of the RPE for Cirrus HD-OCT, as previously mentioned. Given that the BM is on the order of 25 µm thick^18^, based on our study and the works of Tan et al.^5^ and Tepelus et al.^4^, the 15–22-µm difference in CRT between devices that set the outer boundary of the anatomical range at the BM and devices that set it at the middle RPE layer can be explained by the difference in BM thickness.

Furthermore, Sander et al. reported that using two SD-OCT devices (Spectralis and Cirrus HD OCT), CRT measurements provided by the standard software of the instruments differed by 14 µm, whereas those of custom-made software that corrected the anatomic basis from the vitreoretinal surface to the outer border of the RPE did not show a statistically significant difference of ≤ 3 µm^13^. Heussen et al. also reported identical retinal thicknesses following manual correction for the same anatomical basis^12^.

Terasaki et al. compared the foveal microstructure measured by different devices by measuring the four hyperreflective bands of the outer subfovea using three OCT devices (Cirrus HD-OCT, Spectralis, and Topcon 3D OCT-1000 Mark II) and confirmed perfect reproducibility between the devices^19^. It can be assumed that devices that use different algorithms provide the same structural form, and the error in setting the outer boundary of the device used to measure the CRT should have a small effect on the CRT. This suggests that establishing the same anatomical basis is the most important factor for accurate CRT measurement.

This study has several limitations. First, this study includes a relatively small number of subjects. However, it is important to note that the subjects did not have macular disease, high myopia, or advanced age, which affects retinal thickness, and they had relatively the same baseline characteristics. Second, because we did not perform repeated measurements within the same patient to assess measurement-to-measurement differences in this study, we could not confirm the test's repeatability. However, a previous study that measured test repeatability by repeatedly measuring retinal thickness in a sample of subjects demonstrated a fairly high level of reproducibility^3^.

In conclusion, this study aimed to investigate the differences in CRT measurements based on PLEX Elite OCTA, which had not been previously reported, in comparison to other conventional devices. The results showed that CRT values derived from PLEX Elite OCTA exhibited significant differences when compared to some devices. However, there was no significant difference in CRT values when compared to Spectralis, which shares the same anatomical basis, and the correlation was high. Since retinal thickness can be affected by anatomy rather than the image-processing algorithm of the device itself, these differences should be considered when comparing retinal thicknesses measured by different devices.

## Methods

This prospective study was approved by the institutional review board (IRB) of Korea University Medical Center (IRB approval no. 2016AN0344). The data were collected from healthy adults who visited the outpatient clinic for check-ups, starting from May 2017 to August 2018, after obtaining informed consent^8^. All procedures performed in studies involving human participants were in accordance with the ethical standards of the institutional and/or national research committee and with the 1964 Helsinki Declaration and its later amendments or comparable ethical standards. Subjects with high myopia with an axial length of ≥ 26.5 mm; corneal abnormalities; cataracts; vitreous opacities that interfered with image acquisition; and previous retinal disease or treatment with laser, intraocular injections, or surgery were excluded.

Four different devices were used to obtain images, including the Zeiss PLEX Elite (version 1.6.0.21130), Topcon DRI OCT-1 Atlantis (version 10.13.003.06), Zeiss Cirrus 5000 HD-OCT (version 10.0.0.13425), and Heidelberg Spectralis (version 1.10.2.0) systems. The imaging protocol used a volume scan provided by each device to image the central macula to assess retinal thickness according to ETDRS grid zones (central, inner superior, inner temporal, inner inferior, inner nasal, outer superior, outer temporal, outer inferior, and outer nasal). The protocol for each device is described as follows: PLEX Elite, macula thickness analysis with angio 6 × 6 mm (100 kHz) macular scan; DRI OCT-1 Atlantis, 6 × 6 mm 3D macular scan; Cirrus 5000 HD-OCT, 6 × 6 mm with 512 × 128 macular cube; and Spectralis, volume scan of 25 horizontal line scans (512 A-scans per B-scan, 240 µm interscan distance) that covered 20° × 20°, 6 mm × 6 mm centered on the fovea using the automated retinal thickness: high-speed mode). Each of the four devices has different characteristics, and the anatomical basis for measuring CRT differs between them (Table 4)^5^.

If there was poor image quality due to eye motion or blinking artifacts, re-measurement was performed to ensure that the following criteria were met (signal strength, 6/10 with Cirrus 5000 HD-OCT or PLEX Elite; image quality metric, 45/100 with DRI OCT-1 Atlantis; or signal-to-noise ratio, 15/40 dB with Spectralis)^20^. All OCT images were reviewed at the time of acquisition to ensure that the fovea centers were consistent and that the segmentation of the different retinal layers was adequate. Re-measurement in the case of any abnormalities was completed to satisfy these requirements.

To account for the biological bias that can occur when two eyes from the same subject are used, study analysis was performed on the left eye. Each variable was checked for normality of the sample using the Shapiro–Wilk test, and the means of the variables from the four devices were analyzed using the repeated-measures analysis of variance, followed by post hoc testing using Bonferroni correction. Pearson’s correlation coefficient was used to determine the correlation of variables across devices, and the ICC was calculated. SPSS version 20.0 (IBM Corporation, Armonk, NY, USA) was used for statistical analysis, and a *P* < 0.05 was defined as statistically significant.

### Supplementary Information

Supplementary Table S1.

## Data Availability

The raw data from our study is available upon reasonable request from the corresponding author.
