# Supplementary material for: Comparison of retinal thickness measurements among four different optical coherence tomography devices
Source: Sci Rep. 2024 Feb 12;14:3560. doi: 10.1038/s41598-024-54109-6 (PMC10861495; doi:10.1038/s41598-024-54109-6)
Supplement: Supplementary file 1 — Supplementary Table S1. [file 41598_2024_54109_MOESM1_ESM.pdf]

Comparison of Retinal Thickness Measurements among Four Different Optical Coherence Tomography Devices

Ki Tae Nam[1], Cheolmin Yun*[2], Myungho Seo[1], Somin Ahn[2], and Jaeryung Oh[2]

[1]Department of Ophthalmology, Jeju National University College of Medicine, Jeju, Korea

[2]Department of Ophthalmology, Korea University College of Medicine, Seoul, Korea

Supplementary Table S1. Comparison with previous studies measuring central retinal thickness (μm) among different devices

Study	PLEX Elite	Topcon OCT	Cirrus OCT	Spectralis
Current	278.95 ± 20.04	239.57 ± 21.06	256.76 ± 17.82	271.86 ± 17.92
Tan et al [5]		238.0 ± 18.0	254.0 ± 18.1	271.0 ± 18.1
Hanumunthadu et al [9]		239.2 ± 56.1		298.3 ± 70.6
Heussen et al [12]			260.3 ± 19.7	278.9 ± 18.7
Sander et al [13]			269.83 ± 21.50	284.08 ± 20.45
Suzuma et al [14]			382 ± 136	394 ± 139
Matt et al [15]			267 ± 22	284 ± 21
Wolf-Schnurrbusch et al [16]			276 ± 17	288 ± 16
Giani et al [17]		234 ± 35	278 ± 34	292 ± 34

Retinal thickness automatically provided by the device
